# Diagnostic accuracy of the European League against rheumatism/American College of Rheumatology-2019 versus the Systemic Lupus International Collaborating Clinics-2012 versus the ACR-1997 classification criteria in adult systemic lupus erythematosus: A systematic review and meta-analysis

**DOI:** 10.3389/fimmu.2022.1023451

**Published:** 2022-10-12

**Authors:** Wentian Lu, Fengmei Tian, Jinlu Ma, Ying Zhong, Zhichun Liu, Leixi Xue

**Affiliations:** ^1^ Department of Hematology, Huzhou Central Hospital, Affiliated Huzhou Hospital Zhejiang University School of Medicine, Huzhou, China; ^2^ Department of Rheumatology and Immunology, The Second Affiliated Hospital of Soochow University, Suzhou, China; ^3^ Nursing Department, The Second Affiliated Hospital of Soochow University, Suzhou, China

**Keywords:** systemic lupus erythematosus, classification criteria, ACR-1997, SLICC-2012, EULAR/ACR-2019

## Abstract

**Aim:**

To evaluate the diagnostic performance of the American College of Rheumatology (ACR)-1997, the Systemic Lupus International Collaborating Clinics (SLICC)-2012, and the European League against Rheumatism (EULAR)/ACR-2019 classification criteria in adult patients with systemic lupus erythematosus (SLE).

**Methods:**

PubMed, Embase, Web of Science and Cochrane Library databases were searched for literature comparing the three classification criteria of ACR-1997, SLICC-2012 and EULAR/ACR-2019, which took clinical diagnosis as reference. Meta-analysis was used to evaluate and compare the sensitivity, specificity and diagnostic odds ratio of ACR-1997, SLICC-2012 and EULAR/ACR-2019. To assess the early diagnosis capability of the classification criteria, subgroups of patients with disease duration < 3 years and < 1 year were selected for comparison of sensitivity and specificity based on the inclusion of the original study. The sensitivity and specificity of each item in three sets of classification criteria were evaluated. In addition, the clinical and immunological characteristics of patients who did not meet the three classification criteria were compared.

**Results:**

Nine original studies were included in the analysis, including 6404 SLE patients and 3996 controls. Results showed that the diagnostic odds ratios (95% confidence interval) of the SLICC-2012 [136.35 (114.94, 161.75)] and EULAR/ACR-2019 [187.47 (158.00, 222.42)] were higher than those of the ACR-1997 [67.53 (58.75, 77.63)]. Compared with ACR-1997[(0.86 (0.82, 0.89)], SLICC-2012[(0.96 (0.93, 0.97)] and EULAR/ACR-2019[(0.95 (0.92, 0.97)] had higher sensitivity. The specificity of the three classification criteria was similar: ACR-1997, SLICC-2012, and EULAR/ACR-2019 were 0.93 (0.89, 0.95), 0.86 (0.79, 0.91), and 0.91 (0.85, 0.95), respectively. The sensitivity of SLICC-2012 and EULAR/ACR-2019 were higher than that of ACR-1997 in early-course subgroups. Patients who did not meet ACR-1997 had more hypocomplementemia, patients who did not meet SLICC-2012 had more cutaneous lupus and photosensitivity, and patients who did not meet EULAR/ACR-2019 had more cutaneous lupus and leucopenia.

**Conclusions:**

SLICC-2012 and EULAR/ACR-2019 have better diagnostic ability than the ACR-1997, and the sensitivity of the former two criteria is also higher than that of the latter; Moreover, the SLICC-2012 and EULAR/ACR-2019 for patients in the early stages of disease performed equally excellent.

## Introduction

Systemic lupus erythematosus (SLE) is an autoimmune disease with high heterogeneity and multi-system involvement (1, 2). Several international organizations have formulated classification criteria in order to facilitate research, among which the one revised and launched by the American College of Rheumatology (ACR) in 1997, namely ACR-1997, is the most commonly used (3, 4). However, incessant debates on the ACR-1997, such as the redundancy of criteria and the incomplete inclusion of items, had been provoked upon its release (5). As such, a novel classification criteria was designed by the Systemic Lupus International Collaborating Clinics (SLICC) in 2012, called SLICC-2012 (5). Based on the SLICC criteria, a patient is classified as suffering SLE upon meeting four or more clinical and immunologic criteria with at least one clinical criterion and one immunologic one included, or upon getting biopsy-confirmed lupus nephritis with antinuclear antibodies (ANA) or anti-dsDNA antibodies positive; on the contrary, the same case was excluded from the diagnosis of SLE according to the ACR-1997 (3, 4).

In 2019, the European League Against Rheumatism (EULAR) and ACR jointly formulated a new classification standard for SLE, named EULAR/ACR-2019. In this latest standard, ANA of ≥1:80 is deemed a required entry criterion; totally, seven clinical and three immunologic criteria are included, each of which was assigned a weighted score. Patients with a total score of at least 10 can be diagnosed as SLE (6). Its diagnostic performance has been confirmed in some studies (7–14). But there is no consensus on the optimal classification standard in view of significant differences in sensitivity, specificity and accuracy of these three existing criteria for diagnosing SLE patients with different races and ages (15–18). The exploration on the performance of ACR-1997, SLICC-2012, and EULAR/ACR-2019 in a larger heterogeneous patient population will catch their differences, merits and demerits.

As such, we performed a meta-analysis using original studies that verified the performance of ACR-1997, SLICC-2012, and EULAR/ACR-2019 in adult patient with SLE. Since there is no gold standard for diagnosis of SLE, the clinical diagnosis is used as the judgment standard in our study. The study evaluated: 1) sensitivity, specificity, accuracy, positive likelihood ratio, negative likelihood ratio, and diagnostic odds ratio for ACR-1997, SLICC-2012, and EULAR/ACR-2019 classification criteria (**Supplementary Table S1**); 2) the diagnostic ability of the three sets of classification criteria in the early stage of the disease; 3) the sensitivity and specificity of each item in the three sets of classification criteria; 4) the influence of differences in classification criteria on the diagnosis of SLE patients.

## Methods

### Study selection

Systematic searches were conducted in PubMed, Web of Science, Embase, and Cochrane Library on Feb 26, 2022 (**Supplementary Table S2**). Papers were retrieved using the keywords and their synonyms: systemic lupus erythematosus”, “Classification Criteria”, “American College of Rheumatology”, “Systemic Lupus International Collaborating Clinics”, and “European League against Rheumatism”. To obtain articles comparing ACR-1997, SLICC-2012, and EULAR/ACR-2019 on the same populations, only records from 2019 onwards were searched. The inclusion criteria for the original studies in our review: 1) Observational studies (including retrospective cohort studies and case-control studies); 2) Including sensitivity data of ACR-1997, SLICC-2012, and EULAR/ACR-2019 classification criteria. The exclusion criteria: 1) Not in English; 2) No control group; 3) The reference in the diagnosis of the patient was not the separate blinded expert diagnosis. With reference to the separate blinded expert diagnosis used when the EULAR/ACR-2019 classification standard was proposed (6), the clinical diagnosis will continue to be the reference criteria for judging whether the patient has SLE in our study. The titles and abstracts of articles searched by two researchers (Wentian Lu and Fengmei Tian) using the above mentioned keywords were initially screened to obtain all original articles meeting eligibility criteria.

### Data extraction

Data were extracted on first author, year, countries, number of research centers, characteristics of patients and controls (e.g., diagnostic criteria, classification criteria, number, ethnicity, gender proportion, age at diagnosis, disease duration, and so on), sensitivities and specificities of each item for each classification criteria in case and control groups, and missing data on criteria. If raw data on criteria fulfillment, i.e., true positive (TP), false positive (FP), false negative (FN), true negative (TN), was unavailable, the sensitivity and specificity values were used to back-calculate, or vice versa. Jinlu Ma, and Ying Zhong extracted data independently.

### Data analysis

Original articles using clinical diagnosis as diagnostic criteria and evaluating diagnostic abilities for ACR-1997, SLICC-2012, and EULAR/ACR-2019 were included. Calculation of sensitivity and specificity with confidence intervals (CI) for each study was performed using Review Manager 5.4, and summarize receiver operating characteristics (SROC) curves were drawn. R software (version 4.1.2) was used to perform a meta-analysis using a bivariate model recommended in the Cochrane Handbook for Systematic Reviews of Diagnostic Test Accuracy to assess overall sensitivity and specificity (19). The diagnostic odds ratio (DOR) is the ratio of positive likelihood ratio (PLR) to negative likelihood ratio (NLR). The risk of bias and quality of the studies included in meta-analysis were assessed according to the QUADAS-2 form (20).

To determine which classification criteria could classify patients earliest in disease course, the performance in adult patients with disease duration less than 3 years was compared. In addition, sensitivities and specificities of respective criteria items of ACR-1997, SLICC-2012, and EULAR/ACR-2019 were compared in each study. Simultaneously, we compared characteristics of patients meeting one classification criteria but not another two for analyzing how differences among ACR-1997, SLICC-2012, and EULAR/ACR-2019 influence the characteristics of the classified SLE population.

## Results

### Studies included and their characteristics

**Figure 1:**
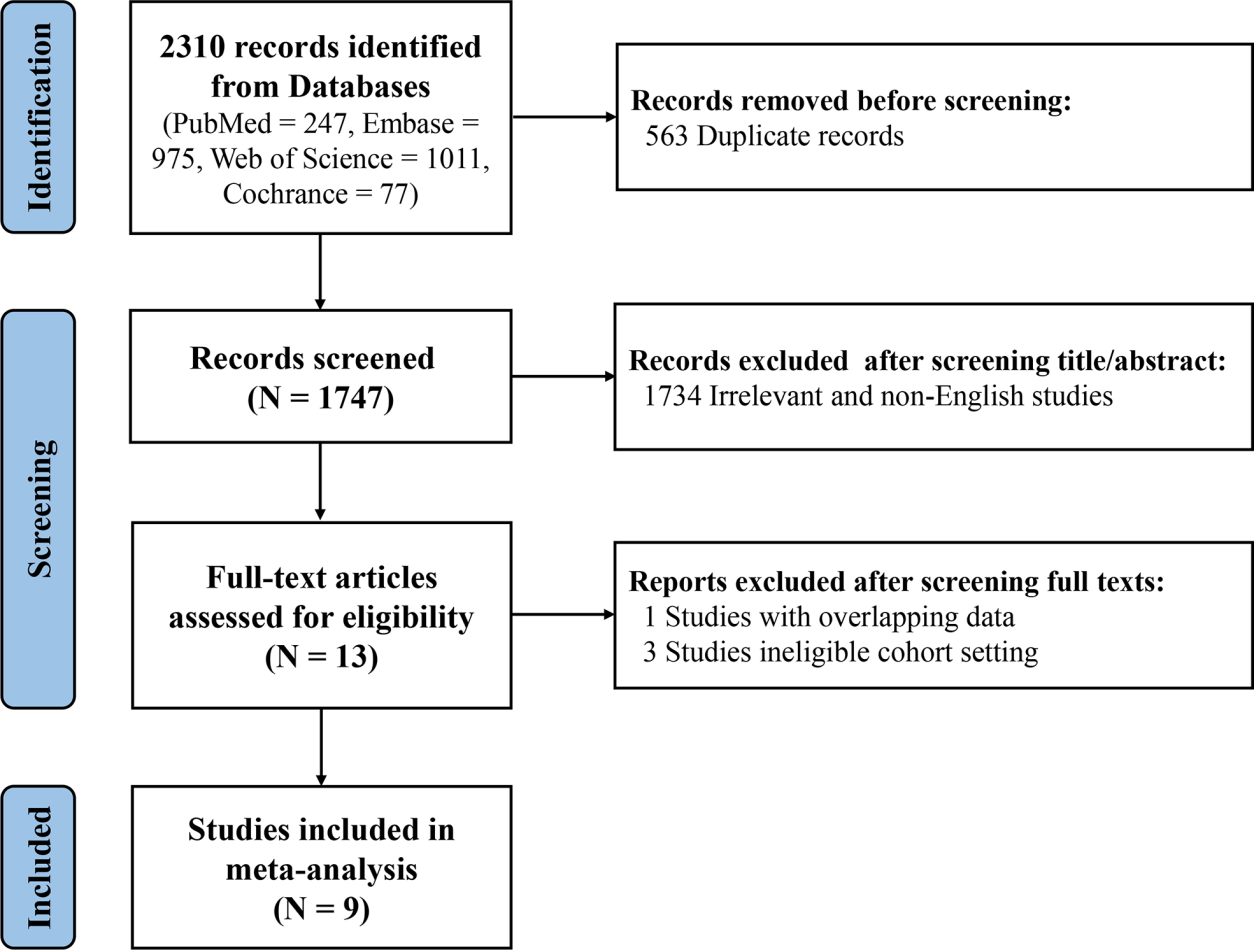
PRISMA 2020 Flow Diagram.

After reviewing the titles and abstracts of 1747 records, 13 studies meeting the inclusion criteria were preliminarily identified. By screening full texts of these eligible studies, one article with the same patient cohort was excluded (21). A study conducted by Dahlström et al. was not included because of using ACR-1982 classification criteria and/or Fries diagnosis criteria as the criteria to distinguish SLE patients from control group (22). Another two studies without setting a control group were excluded as well (23, 24). Ultimately, 9 articles were included in this meta-analysis, comprising 3 European studies (7, 9, 13), 2 north American studies (6, 12) and 4 Asian studies (10, 11, 14, 25). Due to the setting of derivative cohort and validation cohort from different sources in one study (Aringer et al) (6), a total of 10 research cohorts were included in this meta-analysis ultimately. (**Figure 1**) In the aggregate, there were 10,400 subjects with 6,404 SLE patients. Characteristics of each study were shown in **Supplementary Table S3**.

### Methodological quality

**Figure 2:**
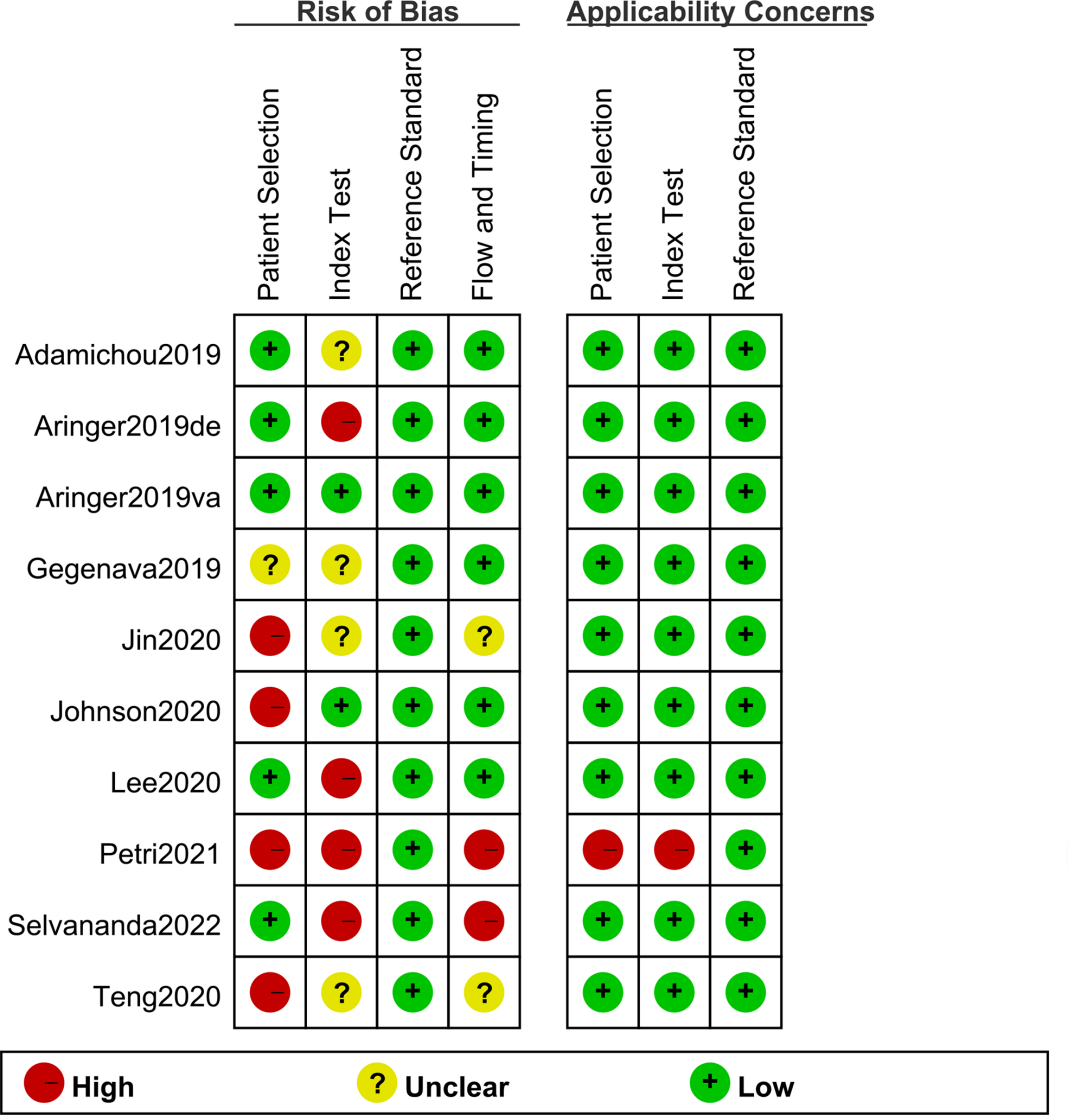
Methodological Quality. Risk of bias and applicability concerns in each study are shown.

A total of nine original studies were included in our systematic review, including five retrospective cohort studies (including six pairs of study cohorts) (6, 7, 9, 10, 14) and four case-control studies (11–13, 25); of them, three studies were conducted under the circumstances that the clinical diagnosis was prior to classification criteria scoring, causing a high risk of bias (10, 12, 14). The scoring of EULAR/ACR-2019 in derivative cohort by Aringer et al. was obtained by rheumatologists without knowing its specific classification threshold, thus its bias risk is rated as “high” (6). In addition, 2 studies scored the three sets of classification criteria at the same time as the clinical diagnosis, which was considered to a high risk of bias (12, 14). All the studies included were assessed for risk of bias and applicability concerns on the QUADAS-2 form as detailed in **Figure 2**. Based on the assessment results of QUADAS-2 form, 0 point was defines as low, 1 as unclear, and 2 as high; and risk of bias was divided into three levels according to the total points (i.e., 0-2 = low, 3-4 = medium, and ≥5 = high). The overall quality assessments of the studies included were medium. The overall quality assessments scores of each study were shown in **Supplementary Table S3**. However, publication bias was not evaluated due to the limited number of original studies.

### Meta-analyses

**Figure 3:**
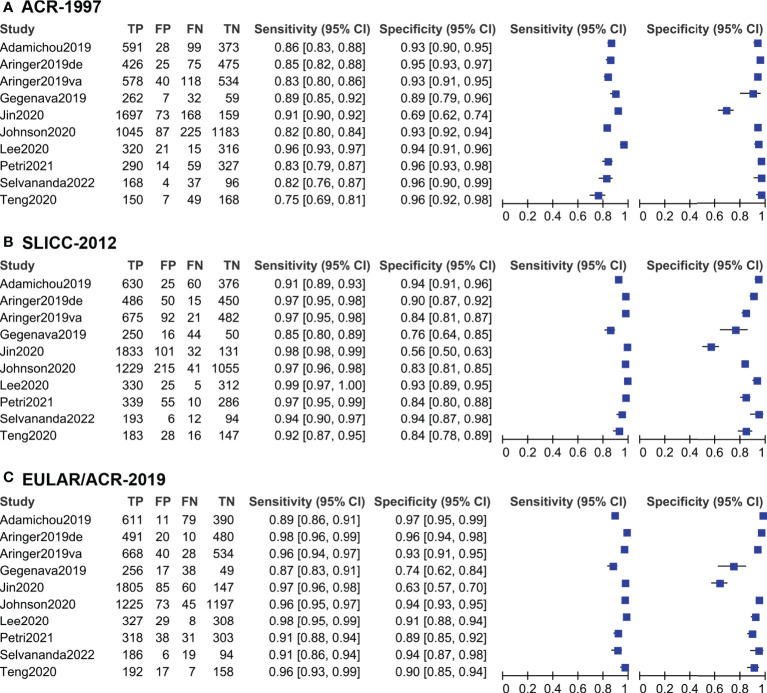
The sensitivity and specificity of ACR-1997 for each study cohort were shown in part **(A)**. And the sensitivity and specificity of SLICC-2012 and EULAR/ACR-2019 were shown in part **(B, C)** respectively. Numbers are rounded off to the nearest integer. Expert diagnosis as the reference standard. TP, true positive; FP, false positive; FN, false negative; TN, true negative. Blue block represents the estimated value, and black line means 95% confidence interval.

**Figure 4:**
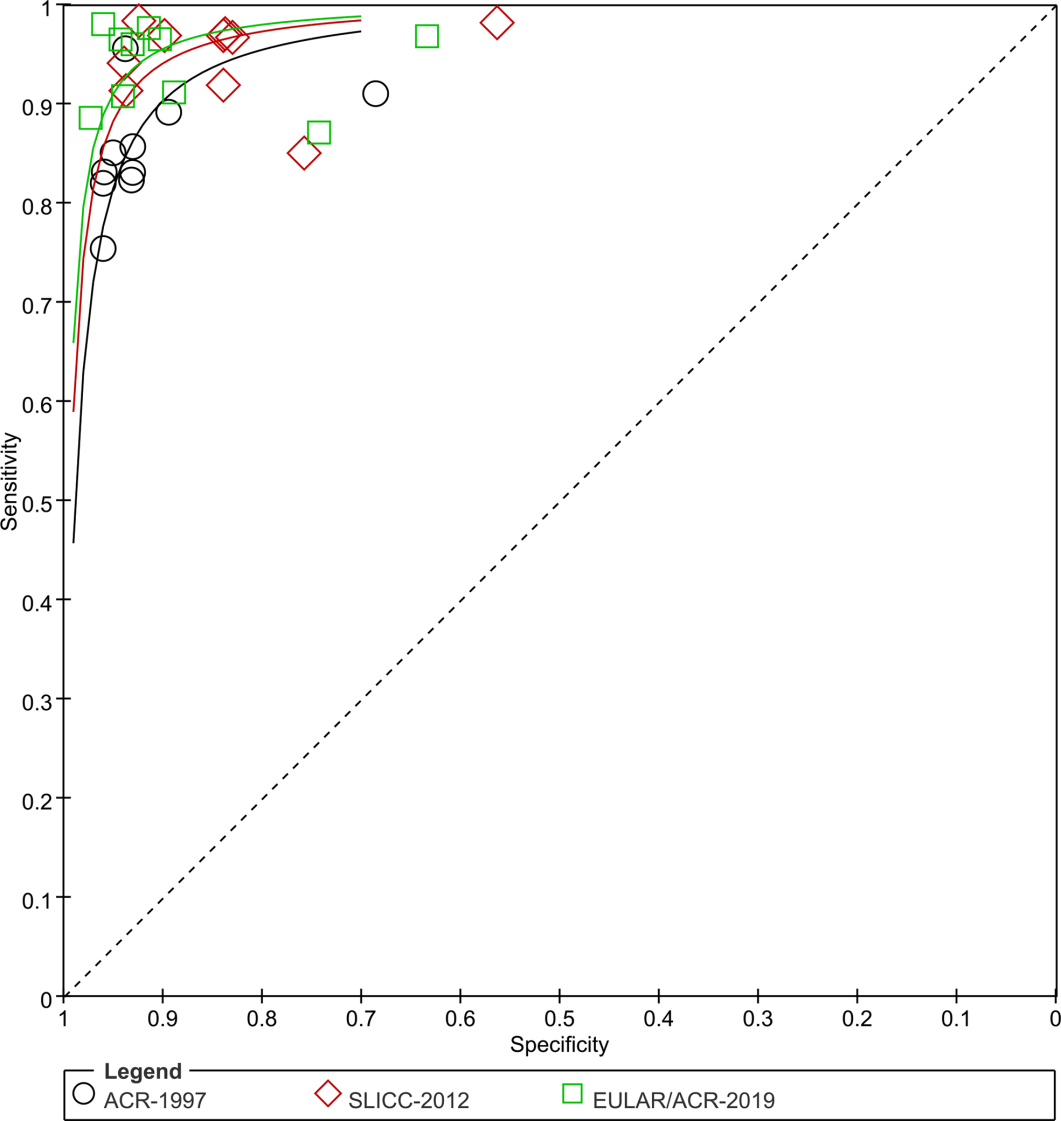
SROC plots. Sensitivity and specificity of all studies are plotted for ACR-1997, SLICC-2012 and EULAR/ACR-2019 (black circles, red diamonds and green blocks). Studies for SLE are numbered: 1 Adamichou 2020 (9), 2 Aringer 2019 (8) (derivation), 3 Aringer 2019 (6) (validation), 4 Gegenava 2019 (7), 5 Jin 2020 (5), 6 Johnson 2020 (13), 7 Lee 2020 (10), 8 Petri 2021 (12), 9 Selvananda 2022 (14), Feb 16, 2022.

The sensitivity and specificity for each study cohort were shown in **Figure 3**. The sensitivities of SLICC-2012 and EULAR/ACR-2019 were higher than that of ACR-1997 in all but one cohorts (7). Results of the meta-analysis over 6,404 SLE and 3,996 control patients were shown in **Table 1**, and SROC plot in **Figure 4**. The DOR (95% CI) of the SLICC-2012 [136.35 (114.94, 161.75)] and EULAR/ACR-2019 [187.47 (158.00, 222.42)] were higher than those of the ACR-1997 [67.53 (58.75, 77.63)]. Compared with ACR-1997 [0.86 (0.82, 0.89)], SLICC-2012 [0.96 (0.93, 0.97)] and EULAR/ACR-2019 [0.95 (0.92, 0.97)] had higher sensitivity. The specificity of the three classification criteria was similar: ACR-1997, SLICC-2012, and EULAR/ACR-2019 were 0.93 (0.89, 0.95), 0.86 (0.79, 0.91), and 0.91 (0.85, 0.95), respectively.

**Table 1:** Results from meta-analysis.

	ACR-1997	SLICC-2012	EULAR/ACR-2019
Sensitivity	0.86 (95% CI 0.82, 0.89)	0.96 (95% CI 0.93, 0.97)	0.95 (95% CI 0.92, 0.97)
Specificity	0.93 (95% CI 0.89, 0.95)	0.86 (95% CI 0.79, 0.91)	0.91 (95% CI 0.85, 0.95)
Positive likelihood ratio	9.87 (95% CI 8.91, 10.94)	5.89 (95% CI 5.48, 6.32)	9.94 (95% CI 9.03, 10.94)
Negative likelihood ratio	0.17 (95% CI 0.16, 0.18)	0.05 (95% CI 0.04, 0.06)	0.01 (95% CI 0.00, 0.01)
Diagnostic odds ratio	67.53 (95% CI 58.75, 77.63)	136.35 (95% CI 114.94, 161.75)	187.47 (95% CI 158.00, 222.42)

### Regional differences

Because only 3 of all the included studies clearly illustrated the racial data, the differences of the sensitivities and specificities of each SLE classification criteria between different races cannot be effectively compared. But each original study described the research centers where the included cases were registered. Accordingly, we compared the differences between the Western and Asian countries (**Table 2**). Consistent with the above results, the sensitivities of SLICC-2012 and EULAR/ACR-2019 were better than ACR-1997 in two regions, and SLICC-2012 was slightly better. But the specificity of ACR-1997 was better than the other two. The sensitivity of them in Asia was higher than that in Europe and the United States, but their specificity was higher in western countries.

**Table 2:** Difference between the Western and Asian countries.

Classification Criteria	Sensitivity (95% CI)	Specificity (95% CI)
Western Countries		
ACR-1997	0.86 [0.84, 0.88]	0.94 [0.92, 0.95]
SLICC-2012	0.91 [0.90, 0.93]	0.88 [0.86, 0.90]
EULAR/ACR-2019	0.89 [0.87, 0.91]	0.92 [0.90, 0.94]
Asian Countries		
ACR-1997	0.90 [0.88, 0.91]	0.88 [0.85, 0.90]
SLICC-2012	0.98 [0.97, 0.98]	0.81 [0.78, 0.84]
EULAR/ACR-2019	0.96 [0.96, 0.97]	0.84 [0.81, 0.86]

### Classification early in disease course

There are 3 studies that evaluated the performance early in the disease course, but the results as well as the definition of “early SLE” were inconsistent (**Table 3**). Adamichou et al. used SLE patients with a course of less than 3 years to compare the early diagnosis ability of classification criteria, demonstrating that the sensitivity of SLICC-2012 and EULAR/ACR-2019 was increased compared with ACR-1997 (91% and 87% vs. 80%) (9). The time interval from the presence of earliest item to meeting the classification criteria was calculated in the same subgroup, and the median time interval of SLICC-2012 and EULAR/ACR-2019 were shorter than that of ACR-1997 (9.1 and 9.1 vs. 12.1 months). Also, Johnson et al. identified the course of disease less than 3 years as early SLE (13). In the subgroup of patients with a course of disease ≥ 1 year but < 3 years, the sensitivity of SLICC-2012 and EULAR/ACR-2019 was higher than that of ACR-1997 (98% *vs*. 97% *vs*. 81%), while the highest specificity was seen in EULAR/ACR-2019 (88% vs. 96% vs. 95%); in subgroup with a course of less than 1 year (including 2 studies), a comparable sensitivity or specificity was observed. It follows that the SLICC-2012 and EULAR/ACR-2019 have a stronger ability to diagnose SLE than the ACR-1997 in the early stage of the disease based on the above two studies. On the contrary, Selvananda et al. reported the classification performance of EULAR/ACR-2019 in patients with a course of no more than 1 year (14). The sensitivity of ACR-1997, SLICC-2012, and EULAR/ACR-2019 were 86%, 98%, and 94%, respectively; the sensitivity of SLICC-2012 was significantly higher than that of ACR-1997; but equivalent specificities of the three were observed.

**Table 3:** Diagnostic ability of ACR-1997, SLICC-2012, and EULAR/ACR-2019 classification criteria in patients with early SLE.

Study	Sensitivity (95%CI)			Specificity (95%CI)		
	ACR-1997	SLICC-2012	EULAR/ACR-2019	ACR-1997	SLICC-2012	EULAR/ACR-2019
Adamichou 2020 (9) DD < 3 years	0.80	0.91	0.87	Not reported	Not reported	Not reported
Johnson2020 (13)						
DD ≥ 1 years and <3years	0.81 (0.72, 0.88)	0.98 (0.93, 1.00)	0.97 (0.92, 0.99)	0.95 (0.88, 0.98)	0.88 (0.80, 0.94)	0.96 (0.90, 0.99)
DD < 1 years	0.56 (0.21, 0.86)	0.89 (0.52, 0.99)	0.89 (0.52, 1.00)	0.92 (0.74, 0.99)	0.92 (0.74, 0.99)	0.92 (0.74, 0.99)
Selvananda2022 (14) DD < 1 years	0.86 (0.79, 0.91)	0.98 (0.94, 0.99)	0.94 (0.88, 0.97)	0.95 (0.86, 0.99)	0.94 (0.83, 0.98)	0.95 (0.86, 0.99)

DD, disease duration.

### Sensitivity and specificity of criteria items

Sensitivities and specificities of each item in respective study cohort were reported in 4 studies (**Supplementary Table S4**) (7, 10, 11, 14). In ACR-1997, items, including malar rash, photosensitivity, arthritis, immunologic, antinuclear antibody (≥1:100), had higher sensitivities, and a good specificity was observed in all items but antinuclear antibody. The items with higher sensitivities in SLICC-2012 were acute cutaneous lupus, synovitis, leukopenia, ANA (≥1:100), anti-dsDNA, low complement, of which acute cutaneous lupus, synovitis, leukopenia, anti-dsDNA, low complement also have good specificities. In EULAR/ACR-2019, 5 items, i.e., entry criterion (ANA≥1:80), leucopenia, acute cutaneous lupus, joint involvement, low C3 or low C4, were reported to not only have sensitivities of more than 50%, but also show better specificities. Of note, the sensitivities of the fever item newly added into EULAR/ACR-2019 were low in all 4 studies, but specificities were better.

Certain items have been observed to differ in their sensitivity and specificity among studies. The positive rate of antiphospholipid antibodies appeared higher in the Dutch neuropsychiatric systemic lupus erythematosus (NPSLE) cohort (48.6%) (7). The prevalence of renal was higher in the Korean cohort from Lee et al. (60.9%) (10). And in the Chinese cohort from Teng et al., a higher incidence of hemolytic anemia was seen (48.0%).

### Characteristics of patients not meeting the three classification criteria

Due to the differences in items of the three classification criteria, there will be a degree of disparity between case groups. Totally, 4 studies reported the characteristics of SLE patients who did not meet the ACR-1997, SLICC-2012, or EULAR/ACR-2019 in the case groups.

In the study by Adamichou et al, patients who were not classified as SLE by ACR-1997 had higher positive rates of hematological diseases and immunological abnormalities, hypocompletemia, direct Coombs test, antinuclear antibody; patients with a higher prevalence of acute cutaneous lupus and synovitis were not classified by SLICC-2012 and those with a higher positive rate of mucocutaneous lesions and leucopenia not done by EULAR/ACR-2019 (9). A Korean study by Lee et al. demonstrated five patients who met SLICC-2012 but lost SLE classification using EULAR/ACR-2019; of them, 3 had a total score of less than 10, and the other 2 patients with lymphopenia (<103/mm3) did not meet leucopenia item (white blood cell count <4.0×109/L) (10). In addition, 2 patients with antinuclear antibody, cutaneous lupus and joint involvement can be classified as SLE by EULAR/ACR-2019 but not by ACR-1997 or SLICC-2012.

In a Chinese study by Teng et al, there were 9 patients only satisfying EULAR/ACR-2019, of which 4 were attributed to fever item; one with oral ulcers, leucopenia, hemolytic anemia, thrombocytopenia and antinuclear antibody can just be classified by SLICC-2012; on the contrary, another one with acute cutaneous lupus, photosensitivity, anti-dsDNA, ANA met ACR-1997 and EULAR/ACR-2019 but not SLICC-2012; notably, a total of 33 patients satisfying both EULAR/ACR-2019 and SLICC-2012 lost SLE classification using ACR-1997 (11). Jin et al. pointed out 132 SLE patients with missed diagnosis by ACR-1997, with a high prevalence of renal disorder, non-scarring alopecia and hypocompletemia; no photosensitivity in SLICC-2012 was accountable for 8 SLE patients who lost SLE classification by SLICC-2012; there were 29 SLE patients not classified by EULAR/ACR-2019, of whom the proportion was higher in negative ANA, mucocutaneous lesions, joint involvement, renal disorder and hematological diseases (25).

## Discussion

SLE is a challenging disease that presents unique issues in diagnosis and treatment. Due to multiple diseases that need to be distinguished and the lack of characteristic biomarkers with high sensitivity, no gold standard for diagnosing SLE is available in clinical practice now, only with some classification criteria to help clinicians make a diagnosis and for scientific research (26–28). The dynamic changes in manifestations of SLE makes any classification criterion very complex. Of significance, classification criteria are utilized to identify distinct subjects suitable for research, thus excluding those uncharacteristic patients (29). The present study demonstrated that SLICC-2012 and EULAR/ACR-2019 are the better SLE classification criterion, with good sensitivity, moderate specificity, and higher DOR values.

In the EULAR/ACR-2019, ANA positive (titer ≥ 1:80) is added as the entry criterion for the classification of SLE. Studies have shown that ANA-negative patients present a low prevalence of renal and neurologic disorders (30). Pisetsky et al. also pointed out that the addition of ANA negative as one item would complicate the screening of SLE patients in clinical trials (31). Despite the report that SLE patients with negative ANA had serious conditions during the disease course, such as nervous system involvement (32, 33), kidney involvement (34), and pulmonary hypertension (35), an Italian study revealed that ANA was highly sensitive to confirmed SLE (36). In the current study, a pretty high sensitivity of ANA (all more than 95%) was observed in four study cohorts. A study evaluated the manifestations of 9,851 patients with SLE, and believed that it was of importance for clinicians to understand the clinical performance of SLE for the final diagnosis in the background of negative ANA (37). The exclusion of ANA-negative patients was considered to be acceptable by EULAR and ACR that proposed the novel classification criteria in 2019 (6), because the classification criterion is not designed for clinical decision-making; that is to say, it cannot be used to judge whether patients who do not fulfill the classification criterion should receive appropriate treatment.

The weighted scoring system newly introduced by EULAR/ACR-2019 reflects the current thinking of the international society on SLE (6). The addition of non-infectious fever item help early identification of SLE. In the setting of type III or IV lupus nephritis with the largest weighted score, a patient can be classified as SLE if ANA is positive at the same time, which further optimizes SLICC-2012 (5).

The increase in sensitivity of SLICC-2012 compared with ACR-1997 seems to be predominantly attributed to the addition of considering positive renal histological examination as a sufficient condition as well as of subacute cutaneous lupus (38) and hypocomplementemia (5). As such, these three items that facilitate increasing sensitivity are still used in EULAR/ACR-2019. A recent study by Wang et al. indicated that EULAR/ACR-2019 yielded a high sensitivity in identifying SLE patients in biopsy-confirmed lupus nephritis (39). Given the risk of bleeding of renal biopsy as an invasive examination, a total of 4 guidelines have proposed the indications for the first renal biopsy (40–46), which not all SLE patients are eligible for.

Of note, a highest specificity of EULAR/ACR-2019 was observed in patients with cutaneous lupus erythematosus (CLE) compared with the two classification criteria previously proposed, according to a study by Stec-Polak et al. (47). Also, the erythrocyte-bound complement activation products may be more sensitive than the detection of serum complement proteins (C3, C4) (48).

In 10 study cohorts of the current meta-analysis, the highest prevalence of kidney disease was observed in the Korean cohort of Lee et al, and the positive rate of anti dsDNA antibody in this cohort was higher than that in other cohorts (10). Reportedly, anti chromatin antibodies, including anti dsDNA, can induce kidney damage in SLE patients (49), which was considered to cause the difference.

In the Dutch NPSLE cohort of Gegenava et al, the positive rate of antiphospholipid antibody was significantly higher than that of other cohorts (7). Despite the unidentified mechanisms of NPSLE (50), studies have shown that NPSLE may be closely related to antiphospholipid syndrome (APS) (51).

In the present study, a higher pooled sensitivity of SLICC-2012 or EULAR/ACR-2019 suggests that the two classification criteria can capture more SLE patients who are classified as incomplete lupus erythematosus (iLE), possible systemic lupus erythematosus, and undifferentiated connective tissue disease by ACR-1997. Notably, iLE patients may be a subgroup of SLE with mild clinical symptoms and relatively stable condition, of whom a few progress slowly to SLE and have good prognosis (52, 53). Usually, patients with iLE are different from those with preclinical diseases, and the latter have autoantibodies but no clinical abnormalities (54).

In the clinical practice, SLE classification criteria enable guiding community doctors to purposefully carry out specialist referral for patients with SLE-related manifestations, and assisting rheumatic immunology specialists in clinical diagnosis and targeted treatment. In order to promptly capture patients with the early-stage disease and initiate early intervention, classification criteria need to have the ability of early identification. According to the results of this study, in the early subgroup (whether the early stage was defined as < 1 year or <3 years), the sensitivity of SLICC-2012 and EULAR/ACR-2019 was better than that of ACR-1997; besides, little difference in the specificity of the three classification criteria was observed. The conclusion, however, may be debatable and deserve some discussion because the sample size of the research cohorts included in this study is small.

Differences of ACR-1997, SLICC-2012, and EULAR/ACR-2019 consist in various aspects, such as the number of items, specific definitions, and scoring weights. Comparison of the SLE patients missed by the three classification criteria, especially in mucocutaneous, musculoskeletal, hematologic and immunology showed that the three sets of criteria could capture the SLE patients with various manifestations. This may also provide more reference for clinical management, because SLE patients with different manifestations may have different response to different therapeutic drugs.

This study has several limitations, as follows. First, we didn’t search in Scopus. That may lead to a certain selection bias in our study. The studies included in the current meta-analysis are retrospective, with an inherent selection bias. Ideally, the test of classification criteria should be carried out in a prospective cohort study consisting of patients who are judged by rheumatologists to be at high risk for SLE. Second, the included studies were mainly conducted in European, American and Asian populations; according to the United Nations 2015 demographic data, this present study covers only 34.4% of the world’s population at a rough estimate (55). Third, due to the lack of consensus on diagnostic criteria for SLE during the study design, clinical diagnosis was determined as the diagnostic gold standard. Given the subjectivity inherent in clinical diagnosis, rheumatologists may be affected by clinical experience, social geographical factors, and the development of classification criteria. Last, the population selection of control group in each original study was different; namely, the control group of 7 studies (8 study cohorts) was basically composed of other rheumatic patients (6, 7, 9–12, 14), one study consisted of patients with cutaneous lupus erythematosus (25), and the composition of the control group in the another one was unknown (13). The above control groups cannot include all diseases that need to be differentiated from SLE in the real world.

It is recommended to refer to the SLICC-2012 or EULAR/ACR-2019 classification standards in clinical practice to improve the accuracy of diagnosis. However, we also encourage clinicians to report and share more data on patients diagnosed by EULAR/ACR-2019 classification standards to further validate its diagnostic performance and promote the emergence of new standard.

To conclude, SLICC-2012 and EULAR/ACR-2019 have better diagnostic ability than the ACR-1997, and the sensitivity of the former two criteria is also higher than that of the latter; Moreover, the SLICC-2012 and EULAR/ACR-2019 for patients in the early stages of disease performed equally excellent.

## Data availability statement

The original contributions presented in the study are included in the article/**Supplementary Material**. Further inquiries can be directed to the corresponding authors.

## Author contributions

WL and FT used the titles and abstracts of the articles searched by the keywords to conduct preliminary screening and obtain all eligible original articles. JM and YZ extracted data together. WL and FT performed part of the statistical analyses. JM and YZ assisted in data analyses. WL and FT drafted the manuscript. LX and ZL reviewed, conceived and supervised the study. WL and FT contributed equally to this work and shared first authorship. All authors contributed to the article and approved the submitted version.

## Funding

This work was supported by the Suzhou Science and Technology Project (SKJY2021098) and Suzhou Gusu health talent plan (2022194).

## Acknowledgments

We thank all the clinicians and nurses at the Department of Rheumatology and Immunology at The Second Affiliated Hospital of Soochow University and the Department of Hematology at Huzhou Central Hospital for their efforts. The systematic review and meta-analysis of the comparison of three classification criteria was made by Lu WT and Tian FM. Both authors were involved in drafting and revising the article and approved the final version to be published.

## Conflict of interest

The authors declare that the research was conducted in the absence of any commercial or financial relationships that could be construed as a potential conflict of interest.

## Publisher’s note

All claims expressed in this article are solely those of the authors and do not necessarily represent those of their affiliated organizations, or those of the publisher, the editors and the reviewers. Any product that may be evaluated in this article, or claim that may be made by its manufacturer, is not guaranteed or endorsed by the publisher.
