# Supplementary material for: Diagnostic accuracy of the European League against rheumatism/American College of Rheumatology-2019 versus the Systemic Lupus International Collaborating Clinics-2012 versus the ACR-1997 classification criteria in adult systemic lupus erythematosus: A systematic review and meta-analysis
Source: Front Immunol. 2022 Oct 12;13:1023451. doi: 10.3389/fimmu.2022.1023451 (PMC9599400; doi:10.3389/fimmu.2022.1023451)
Supplement: Supplementary file 1 [file Table_1.docx]

**Supplementary Table S1. Glossary**

| **Term** | **Abbreviations** | **Calculation Formula/ paraphrase** |
| --- | --- | --- |
| true positive | TP | Diagnosed as SLE by classification criteria, and clinically diagnosed as SLE |
| false positive | FP | Diagnosed as SLE by classification criteria, but not clinical diagnosed as SLE |
| false negative | FN | Not diagnosed as SLE by classification criteria, but clinically diagnosed as SLE |
| true negative | TN | Not diagnosed as SLE by classification criteria, and not clinical diagnosed as SLE |
| Sensitivity | Sen | TP/(TP+FN) |
| Specificity | Spe | TN/(TN+FP) |
| Accuracy | ACC | (TP+TN)/(TP+FP+FN+TN) |
| Positive likelihood ratio | PLR | Sen/(1-Spe)=[TP/(TP+FN)]/[FP/(TN+FP)] |
| Negative likelihood ratio | NLR | (1-Sen)/Spe=[FN/(TP+FN)]/[TN/(TN+FP)] |
| Diagnostic odds ratio | DOR | PLR/NLR=(TP*TN)/(FP*FN) |

**Supplement Table S2. Additional information on search strategy**

**Table S1A. PubMed 247 results (Feb 16, 2022)**

| **No.** | **Query** | **Results** |
| --- | --- | --- |
| #5 | #1 AND #2 AND #3 AND #4 | 247 |
| #4 | ("2019"[Date - Publication] : "2022"[Date - Publication]) | 4,568,267 |
| #3 | (((European League against Rheumatism[Title/Abstract]) OR (EULAR[Title/Abstract])) OR (American College Rheumatology[Title/Abstract])) OR (ACR[Title/Abstract]) | 14,240 |
| #2 | (criteria[Title/Abstract]) OR (classification[Title/Abstract]) | 997,863 |
| #1 | ((SLE[Title/Abstract]) OR (systemic lupus erythematosus[Title/Abstract])) OR (lupus[Title/Abstract]) | 89,090 |

**Table S1B. Embase 975 results (Feb 16, 2022)**

| **No.** | **Query** | **Results** |
| --- | --- | --- |
| #5 | #1 AND #2 AND #3 AND [2019-2022]/py | 975 |
| #4 | #1 AND #2 AND #3 | 3,961 |
| #3 | 'european league against rheumatism':ab,ti OR eular:ab,ti OR 'american college rheumatology':ab,ti OR acr:ab,ti | 37,059 |
| #2 | criteria:ab,ti OR classification:ab,ti | 1,516,130 |
| #1 | sle:ab,ti OR 'systemic lupus erythematosus':ab,ti OR lupus:ab,ti | 128,844 |

**Table S1C. Web of Science 1011 results (Feb 16, 2022)**

| **No.** | **Query** | **Results** |
| --- | --- | --- |
| #5 | #1 AND #2 AND #3 and 2019 or 2020 or 2021 or 2022 (Publication Years) | 1,011 |
| #4 | #1 AND #2 AND #3 | 3,565 |
| #3 | (((TS=(European League against Rheumatism)) OR TS=(EULAR)) OR TS=(American College Rheumatology)) OR TS=(ACR) | 32,664 |
| #2 | (TS=(criteria)) OR TS=(classification) | 2,977,581 |
| #1 | ((TS=(systemic lupus erythematosus)) OR TS=(SLE)) OR TS=(lupus) | 173,721 |

**Table S1D. Cochrane Library 77 results (Feb 16, 2022)**

| **No.** | **Query** | **Results** |
| --- | --- | --- |
| #4 | #1 AND #2 AND #3, Custom Range: 2019-2022 | 77 |
| #3 | (European League against Rheumatism):ti,ab,kw OR (EULAR):ti,ab,kw OR (American College Rheumatology):ti,ab,kw OR (ACR):ti,ab,kw | 7632 |
| #2 | (criteria):ti,ab,kw OR (classification):ti,ab,kw | 292737 |
| #1 | (SLE):ti,ab,kw OR (systemic lupus erythematosus):ti,ab,kw OR (lupus):ti,ab,kw | 3813 |

**Supplementary Table S3. Study characteristics**

| **General study information** | | | | | | **Case characteristics : Control characteristics** | | | | | | **Reason for exclusion from meta-analysis** | **The overall quality assessments score** | **Risk of bias** |
| --- | --- | --- | --- | --- | --- | --- | --- | --- | --- | --- | --- | --- | --- | --- |
| **First author** | **Time** | **type of studies** | **Setting (nr. of centres, setting, country)** | **Reference** | **Index** | **N** | **%F** | **AaD** | **DD** | **Ethnicity** | **Control group patients** |  |  |  |
| Adamichou (9) | 2019 | A retrospective cohort study | Two centers from Greece | Separate blinded expert diagnosis | ACR-1997、SLICC-2012、EULAR/ACR-2019 | 690 : 401 | NR : NR | NR : NR | NR : NR | NR : NR | Other rheumatological diseases (n=401) | - | 1 | Low |
| Aringer (De) (6) | 2019 | A retrospective cohort study | Twenty-one centers from the US, Canada, Mexico, Austria, Croatia, France, Germany, Greece, Hungary, Italy, Portugal, Spain, the UK, Turkey, Hong Kong, and Japan | Separate blinded expert diagnosis | ACR-1997、SLICC-2012、EULAR/ACR-2019 | 501 : 500 | 89 : 84 | 45±14 : 54±16 | 11±8 : 9±8 (years) | Black, East Asian, Hispanic, South/Southeast Asian, White and other : Black, East Asian, Hispanic, South/Southeast Asian, White and other | AOSD(n=2), AIT(n=6), BD(n=7), Cancer(n=2), Inflammatory myositis(n=37), FS(n=6), Membranous nephritis(n=11), MCTD(n=9), OA(n=2), APS(n=45), PA(n=12), RA(n=94), Sarcoidosis(n=2), SS(n=112), SpA(n=5), SSc(n=99), UCTD(n=16), Vasculitis(n=9), Viral infection(n=5), other(n=19), | - | 2 | Low |
| Aringer (Va) (6) | 2019 | A retrospective cohort study | Twenty-one centers from the US, Canada, Mexico, Austria, Croatia, France, Germany, Greece, Hungary, Italy, Portugal, Spain, the UK, Turkey, Hong Kong, and Japan | Separate blinded expert diagnosis | ACR-1997、SLICC-2012、EULAR/ACR-2019 | 696 : 574 | 87 : 85 | 45±14 : 56±16 | 11±8 : 9±8 (years) | Black, East Asian, Hispanic, South/Southeast Asian, White and other : Black, East Asian, Hispanic, South/Southeast Asian, White and other | AOSD(n=11), AIT(n=5), BD(n=9), Cancer (n=9), Inflammatory myositis (n=27), FS (n=3), Membranous nephritis (n=14), MCTD (n=15), APS (n=48), PA (n=9), RA (n=110), Sarcoidosis (n=2), SS (n=124), SpA (n=5), SSc (n=112), TB (n=2), UCTD (n=20), Vasculitis (n=13), Viral infection (n=5), other (n=29) | - | 0 | Low |
| Dahlström (22) | 2019 | A retrospective cohort study | One center from Sweden | ACR-1982 classification criteria and/or Fries classification criteria | ACR-1997、SLICC-2012、EULAR/ACR-2019 | 55 : 56 | 79 : 87 | 49.7 (mean), range 22-81 : 53.5(mean), range 23-88 | NR : NR | NR : NR | pSS(n=12),UCTD(n=8), APS(n=7), RA(n=4), SLE(n=4), FS(n=4), arthralgia (n = 3), PA(n=2), unspecified arthritis (n=2), AOSD(n=1), PM(n=1), SSc with PBC(n=1), MCTD(n=1), PAPA(n=1), RI(n=1), MS(n=1), PR(n=1) and recurrent pleuritis (n = 1) | Reference | - |  |
| Gegenava (7) | 2019 | A retrospective cohort study | One center from the Netherlands | Separate blinded expert diagnosis | ACR-1997、SLICC-2012、EULAR/ACR-2019 | 294 : 66 | 87 : 83 | 43±14 : 46±15 | NR : NR | NR : NR | SLE-like disease(n=20), UCTD(n=12), pSS(n=11), MCTD(n=8), other (including chilblain LE, CDLE, SCLE, APS, DM, CREST, JIA, Behçet-like disease and somatoform disorder ) (n=15) | - | 2 | Low |
| Jin (25) | 2020 | A case-control study | One center from China | Separate blinded expert diagnosis | ACR-1997、SLICC-2012、EULAR/ACR-2019 | 1865 : 232 | 91 : 68 | 31.13±13.10 : 33.88±14.05 | 58.60±67.06 : 53.64±67.27 (months) | NR : NR | CLE(n=232) | - | 4 | Medium |
| Johnson (13) | 2020 | A case-control study | Twenty-one centers from Austria, Canada, Croatia, France, Germany, Greece, Hong Kong, Hungary, Japan, Italy, Mexico, Portugal, Spain, Turkey, UK and USA | Separate blinded expert diagnosis | ACR-1997、SLICC-2012、EULAR/ACR-2019 | 1270 : 1270 | 86 : 86 | NR : NR | NR : NR | white, black, Hispanic (Latin American heritage) and Asian : white, black, Hispanic (Latin American heritage) and Asian | Mimicking controls (details unknown) (n=1270) | - | 2 | Low |
| Lee (10) | 2020 | A retrospective cohort study | One center from South Korea | Separate blinded expert diagnosis | ACR-1997、SLICC-2012、EULAR/ACR-2019 | 335 : 337 | 90 : 87 | 32.0 (median), IQR 19.0 : 48.0 (median), IQR 24.0 | NR : NR | NR : NR | RA(n=92), APS(n=57), MCTD(n=52), SSc(n=43), pSS(n=39), UCTD(n=28), RA with secondary SS(n=24), DM(n=1) and SpA(n=1) | - | 2 | Low |
| Magallares (23) | 2021 | A retrospective cohort study | One center from Switzerland | Separate blinded expert diagnosis | ACR-1997、SLICC-2012、EULAR/ACR-2019 | 79 : - | 89 : - | 51.89±14.04 : - | 15.22±11.59 : - (years) | NR : - | - | No control group | - |  |
| Petri (12) | 2021 | A case-control study | One center from the US | Separate blinded expert diagnosis | ACR-1997、SLICC-2012、EULAR/ACR-2019 | 349 : 341 | NR : NR | NR : NR | NR : NR | NR : NR | RA(n=119), myositis(n=55), CCLE(n=50), UCTD(n=44), vasculitis(n=37), APS(n=33), SSc(n=28), FS(n=25), SS(n=15), rosacea(n=8), psoriasis(n=7), sarcoidosis(n=1) and JIA(n=1) | - | 6 | High |
| Selvananda (14) | 2022 | A retrospective cohort study | One center from Malaysia | Separate blinded expert diagnosis | ACR-1997、SLICC-2012、EULAR/ACR-2019 | 205 : 100 | 88 : 85 | 32.8±12.89 : 44.1±13.5 | 16 (median), IQR 42 : 39 (median), IQR 98 (weeks) | Malay, Chinese, Indians, and others : Malay, Chinese, Indians, and others | RA(n=33), UCTD(n=24), APS(n=6), PM(n=2), SSc(n=9), pSS(n=9), MCTD(n=3), SV(n=5), SSA(n=6), gout (n=1), AOSD(n=1), FS(n=1) | - | 4 | Medium |
| Suda (24) | 2020 | A case-control study | One center from Japan | Separate blinded expert diagnosis | ACR-1997、SLICC-2012、EULAR/ACR-2019 | 100 : - | 91 : - | 44.5 (mean), range 18-88 : - | 135.2 (mean), range 0-549 : - (months) | Asian, Caucasian and Hispanic : - | - | No control group | - |  |
| Teng (11) | 2020 | A case-control study | One center from China | Separate blinded expert diagnosis | ACR-1997、SLICC-2012、EULAR/ACR-2019 | 199 : 175 | 91 : 78 | 37.7±15.9 : 53.75±15.36 | 10.7±14.6 : 24.6±26.8 (months) | NR : NR | RA(n=68), SSc(n=13), pSS(n=54), DM(n=36), lymphoma (n=4) | - | 4 | Medium |

Abbreviations: %F = percentage female participants, AaD = age at diagnosis (in years, mean ±sd), AIT = autoimmune thyroiditis, AOSD = adult-onset Still's disease, APS = antiphospholipid syndrome, BD = Behçet disease, CCLE = chronic cutaneous lupus erythematosus, CDLE = chronic discoid lupus erythematosus, CLE = cutaneous lupus erythematosus, CREST = calcinosis, Raynaud's syndrome，esophageal dysmotility, sclerodactyly and telangiectasis, Da = derivation cohort, DD = disease duration, (mean ±sd), DM = dermatomyositis, FS = fibromyalgia syndrome, IQR = interquartile range, JIA = juvenile idiopathic arthritis, MCTD = mixed connective tissue disease, MS = multiple sclerosis, N = number of participants, NR = not reported, OA = osteoarthritis, PA = psoriatic arthritis, PAPA = pyogenic arthritis, pyoderma gangrenosum and acne syndrome, PBC = primary biliary cirrhosis, PM = polymyositis, PR = palindromic rheumatism, pSS = primary Sjögren's syndrome, RA = rheumatoid arthritis, RI = renal infarction, SCLE = subacute cutaneous 1upus erythematosus, SLE = systemic lupus erythematosus, SpA = spondyloarthropathy, SSA = seronegative spondyloarthropathy, SSc = systemic sclerosis, SV = systemic vasculitis, TB = tuberculosis, UCTD = undifferentiated connective tissue disease, Va = validation cohort.

**Supplementary Table S4. Sensitivity and specificity of ACR-1997, SLICC-2012, and EULAR/ACR-2019 criteria items.**

|  | **Sensitivity (%)** | | | | **Specificity (%)** | | | |
| --- | --- | --- | --- | --- | --- | --- | --- | --- |
|  | **Gegenava**  **2019** | **Lee**  **2020** | **Selvananda**  **2022** | **Teng**  **2020** | **Gegenava**  **2019** | **Lee**  **2020** | **Selvananda**  **2022** | **Teng**  **2020** |
| **ACR-1997** |  |  |  |  |  |  |  |  |
| Malar rash | 43.9 | 63.3 | 51.7 | 23.0 | 86.4 | 96.4 | NR | NR |
| Discoid rash | 17.7 | 7.5 | 18.7 | 30.0 | 89.4 | 100.0 | NR | NR |
| Photosensitivity | 52.7 | 26.0 | NR | 12.0 | 66.7 | 96.4 | NR | NR |
| Oral ulcers | 42.9 | 36.1 | 35.0 | 21.0 | 71.2 | 95.5 | NR | NR |
| Nonerosive arthritis | 62.2 | 65.7 | 49.3 | 62.0 | 83.3 | 68.8 | NR | NR |
| Serositis | 25.9 | 28.4 | NR | 21.0 | 97 | 98.2 | NR | NR |
| Renal | 29.9 | 60.9 | 24.6 | 30.0 | 95.5 | 97.9 | NR | NR |
| Neurologic | 12.6 | NR | NR | NR | 90.9 | NR | NR | NR |
| Hematologic | 49.3 | NR | NR | NR | 84.8 | NR | NR | NR |
| Immunologic | 75.2 | NR | NR | NR | 69.7 | NR | NR | NR |
| ANA | 96.3 | 98.8 | 96.6 | 100.0 | 24.2 | 27.6 | 48.0 | 30.3 |
| **SLICC-2012** |  |  |  |  |  |  |  |  |
| Acute cutaneous lupus | 50.3 | 63.3 | 51.7 | 26.0 | 81.8 | 96.4 | NR | NR |
| Chronic cutaneous lupus | 17.7 | 7.5 | 18.7 | 26.0 | 89.4 | 100.0 | NR | NR |
| Oral ulcers | 42.9 | 36.1 | 35.0 | 19.0 | 71.2 | 95.5 | NR | NR |
| Nonscarring alopecia | 15.0 | 42.7 | 36.0 | 16.0 | 97 | 97.3 | NR | NR |
| Arthritis/ synovitis | 62.2 | 65.7 | 49.3 | 55.0 | 83.3 | 68.8 | NR | NR |
| Serositis | 25.9 | 28.4 | NR | 19.0 | 99 | 98.2 | NR | NR |
| Renal | 29.9 | 60.9 | 24.6 | 31.0 | 95.5 | 97.9 | NR | NR |
| Neurologic | NR | 15.2 | NR | NR | NR | 96.1 | NR | NR |
| Hemolytic anemia | 14.6 | 34.3 | 12.8 | 48.0 | 98.5 | 98.8 | NR | NR |
| Leukopenia | 47.6 | NR | 42.9 | 65.0 | 77.3 | NR | NR | NR |
| Lymphopenia | 19.7 | 39.4 | 22.7 | 30.0 | 93.9 | 93.4 | NR | NR |
| Thrombocytopenia | 96.3 | 98.8 | 96.6 | 100.0 | 24.2 | 27.6 | 48.0 | 30.3 |
| ANA | 57.7 | 89.3 | 53.7 | 86.0 | 77.3 | 90.5 | 94.0 | 96.6 |
| Anti-dsDNA | 12.2 | 26.0 | 20.5 | 25.0 | 97 | 97.9 | 98.0 | 100.0 |
| Anti-Sm | 48.6 | NR | 17.6 | 28.6 | 74.2 | NR | 92.0 | 99.4 |
| Antiphospholipid antibody | 57.1 | 84.7 | 68.3 | 83.0 | 78.8 | 97.0 | 92.0 | 82.9 |
| Low complement | 7.1 | NR | NR | 48.0 | 100 | NR | NR | NR |
| **EULAR/ACR-2019** |  |  |  |  |  |  |  |  |
| ANA | 96.3 | 98.8 | 96.6 | 100.0 | 24.2 | 72.4 | 48.0 | 30.3 |
| Fever | 24.2 | 31.3 | 36.5 | 39.0 | 83.3 | 98.2 | NR | NR |
| Leucopenia | 40.3 | NR | 42.9 | 62.0 | 84.8 | NR | NR | NR |
| Thrombocytopenia | 19.7 | 39.4 | 22.7 | 28.0 | 93.9 | 93.4 | NR | NR |
| Autoimmune haemolysis | 14.6 | 34.3 | 12.8 | 45.0 | 98.5 | 98.8 | NR | NR |
| Delirium | 2.7 | NR | 0.0 | 0.5 | 100 | NR | NR | NR |
| Psychosis | 4.1 | NR | 2.5 | 0.5 | 97 | NR | NR | NR |
| Seizure | 9.5 | NR | 4.9 | 0.5 | 97 | NR | NR | NR |
| Non-scarring alopecia | 15 | 42.7 | 36.0 | 16.0 | 97 | 97.3 | NR | NR |
| Oral ulcers | 42.9 | 36.1 | 35.0 | 17.0 | 71.2 | 95.5 | NR | NR |
| Subacute cutaneous or discoid lupus | 32 | 7.5 | 18.7 | 24.0 | 74.2 | 100.0 | NR | NR |
| Acute cutaneous lupus | 50.3 | 63.3 | 51.7 | 23.0 | 81.8 | 96.4 | NR | NR |
| Pleural or pericardial effusion | 19.8 | NR | NR | 14.0 | 98.5 | NR | NR | NR |
| Acute pericarditis | 11.6 | NR | 0.0 | 2.0 | 98.5 | NR | NR | NR |
| Joint involvement | 62.2 | 65.7 | 49.3 | 54.0 | 83.3 | 68.8 | NR | NR |
| Proteinuria | 22.4 | 60.9 | 24.6 | 31.0 | 98.5 | 97.9 | NR | NR |
| Class II or V lupus nephritis | 10.5 | 33.4 | 2.5 | 1.0 | 97 | 99.7 | NR | NR |
| Class III or IV lupus nephritis | 12.6 |  | 6.0 | 2.0 | 100 |  | NR | NR |
| Antiphospholipid antibodies | 48.6 | NR | 17.6 | 28.6 | 74.2 | NR | 92 | 99.4 |
| Low complement | 57.3 | 84.7 | 68.3 | 77.9 | 78.5 | 97.0 | 92 | 82.9 |
| Anti-dsDNA OR anti-Sm | NR | NR | NR | NR | NR | NR | NR | NR |

Abbreviations: NR = not reported.
